# Socioeconomic status, mental wellbeing and transition to secondary school: Analysis of the School Health Research Network/Health Behaviour in School‐aged Children survey in Wales

**DOI:** 10.1002/berj.3616

**Published:** 2020-03-12

**Authors:** Graham F. Moore, Rebecca E. Anthony, Jemma Hawkins, Jordan Van Godwin, Simon Murphy, Gillian Hewitt, G. J. Melendez‐Torres

**Affiliations:** ^1^ Cardiff University UK; ^2^ University of Exeter UK

**Keywords:** transition, wellbeing, mental health, inequalities

## Abstract

Young people's wellbeing is often lowest where they assume a relatively low position within their school's socioeconomic hierarchy, for example, among poorer children attending more affluent schools. Transition to secondary school is a period during which young people typically enter an environment which is more socioeconomically diverse than their primary school. Young people joining a school with a higher socioeconomic status intake relative to their primary school may assume a relatively lowered position within their school’s socioeconomic hierarchy, experiencing a detriment to their wellbeing as a consequence. This article draws on data from 45,055 pupils in Years 7 and 8, from 193 secondary schools in Wales, who completed the 2017 Student Health Research Network (SHRN) Student Health and Wellbeing (SHW) survey. Pupils reported which primary school they previously attended, and survey data on wellbeing were linked to publicly available data on the free school meal entitlement of schools attended. In cross‐classified linear mixed‐effects models, with primary and secondary school as levels, mental wellbeing varied significantly according to both primary and secondary school attended. A higher school‐level deprivation was associated with worse mental wellbeing in both cases. Mental wellbeing was significantly predicted by the relative affluence of a child's primary and secondary school, with movement to a secondary school of higher overall socioeconomic status associated with lowered wellbeing. These findings highlight transition to secondary school as a key point in which socioeconomic inequality in wellbeing may widen, and thus as an important focal point for intervention to reduce health inequalities.

## Introduction

Numerous indicators of health—such as disability‐free life years, self‐rated health, subjective wellbeing and life expectancy—improve as socioeconomic status (SES) increases (Marmot *et al.*, 2010). This socioeconomic patterning emerges during childhood and adolescence, before widening throughout the life‐course (Hanson & Chen, 2007; Moore *et al.*, 2015; Viner *et al.*, 2015). Adolescence is a particularly critical period for mental wellbeing, with lower wellbeing in adolescence associated with psychosocial difficulties which endure into adulthood (Park, 2004). Mental health and mental wellbeing have some conceptual overlap, but are often considered distinct constructs; wellbeing is typically framed in more positive terms and focused on holistic aspects of young people’s happiness with their lives, by contrast to definitions of mental health which focus on the presence or absence of illness symptoms (Greenspoon & Saklofske, 1998, 2001; Keyes, 2006). Nevertheless, positively framed measures of wellbeing are often similarly predictive of outcomes such as depression, as are symptom‐focused measures (Mukuria *et al.*, 2016). There is international evidence that adolescent health inequalities have recently widened in line with growing international economic inequality (Elgar *et al.*, 2015; Shackleton *et al.*, 2016), while in the UK, young people’s mental health and wellbeing have recently been described as being at crisis point (Gunnell *et al.*, 2018; Taylor‐Robinson *et al.*, 2019). Identifying and influencing risk and protective factors associated with socioeconomic inequalities in adolescent wellbeing is therefore a UK (HM Government, 2010), and international, policy priority (World Health Organization, 2014).

## The role of schools in reducing or increasing inequalities in wellbeing

While one of many interconnected social influences on young people’s health and development (Moore *et al.*, 2018), schools are commonly viewed as a channel for delivery of interventions to reduce childhood inequalities. This is largely due to their capacity to reach whole populations, but while some school‐based actions reduce inequality, others increase it (Moore *et al.*, 2015). A substantial body of the literature demonstrates socioeconomic inequalities in educational attainment, with children from poorer backgrounds, and attending schools with more deprived socioeconomic compositions, achieving less positive educational outcomes overall (Lupton & Thomson, 2015; Francis *et al.*, 2017; von Stumm *et al.*, 2019). Education systems throughout the UK, though particularly in England, have moved towards increasing marketisation in recent decades. Some argue that moves such as the introduction of school choice through the Education Act (1988) increased social stratification within the education system, resulting in increased social segregation (Allen, 2007) and a reproduction of class inequalities (Ball *et al.*, 1996). Theorists such as Basil Bernstein have explained inequalities in education by highlighting a number of mechanisms through which schools act to reproduce or even legitimise inequalities present within wider society, commonly reflecting a more natural extension of the middle‐class family environment (Bernstein, 1975).

However, while well researched in education, the processes through which inequalities in *health and wellbeing* are perpetuated, or mitigated, by the actions of schools are only beginning to receive significant attention. Our analyses of 2009 Health Behaviour in School‐aged Children (HBSC) survey data in Wales demonstrated independent and interacting associations of family affluence and the socioeconomic composition of a child’s school in predicting a range of health behaviours (Moore & Littlecott, 2015). Lower family affluence and higher school‐level deprivation independently predicted a range of risk and protective behaviours, with health ‘benefits’ of attending a more affluent school experienced disproportionately by young people who were also from relatively affluent families (Moore & Littlecott, 2015). Hence, relative social status by comparison to peers, rather than just the affluence of the pupil’s family background in absolute terms, appeared important in shaping health and wellbeing outcomes. These processes are consistent with the ‘frog‐pond effect’ in relation to educational inequalities, whereby young people from poorer backgrounds benefit least from attending a more affluent school in terms of educational outcomes (Bachman & O'Malley, 1986).

In this journal (Moore *et al.*, 2017), we subsequently extended our earlier analyses through the lens of Markham and Aveyard’s (2003) theory of school organisation and human functioning. This theory is informed by a combination of Aristotelian notions of human functioning and the aforementioned work of Bernstein (1975). Locating Aristotelian perspectives within this more socially driven framing, individuals are thought to be in a position to *choose* behaviours supportive of health and wellbeing when capacity for practical reasoning (i.e. viewing problems and solutions from different perspectives) and affiliation (i.e. shared values and empathetic understanding of others’ perspectives) are supported by the social environment provided by schools. Markham and Aveyard (2003) therefore situate the development of these human capacities within the context of schools to theorise how institutional processes enable or constrain a student’s realisation of these potentials through the instructional order (the means of developing knowledge and skills) and the regulatory order (institutional norms, value and belief system). In combining these perspectives, Markham and Aveyard locate notions of human functioning within social structure, by stark contrast to the dominant tendency for use of more individualistic psychological models in school health (Moore *et al.*, 2015). Hence, attention is drawn to schools’ everyday institutional processes, and how these create a social context in which pupils are likely to feel an enhanced, or diminished, sense of connectedness to their school community. It has been used within effective recent interventions which aim to change the culture and climate of school settings through the use of local data and involvement of pupils, teachers and other school staff in decision‐making, to create social environments which better engage pupils with school and improve their wellbeing (Bonell *et al.*, 2018).

Markham and Aveyard (2003) argue that when students are alienated from the instructional order, the regulatory order, or both, they may become disconnected from the learning environment and the values of the school, diminishing opportunities to develop practical reasoning and affiliations. However, while Markham and Aveyard did not explicitly theorise how the organisation of schools may generate or reduce inequality, as theorised by Bernstein, students’ socioeconomic background may significantly influence the nature of their interactions with the instructional and regulatory order. Students from poorer backgrounds may therefore be more likely to become alienated, and distanced from the norms and pedagogical transmission of their school, due to the socially isolating experience of poverty and the incongruence between the values of school, family and other aspects of life (Markham & Aveyard, 2003; Fletcher & Bonell, 2013). Further, a review of qualitative studies of the lived experience of poverty and its impacts on young people’s experiences of schooling concluded that their ‘narratives of school life were often infused with anxiety, uncertainty and a sense of unfairness’, leading to insecure social integration and work to ‘hide’ poverty from peers and school staff (Ridge, 2011), with schools therefore becoming a stressful environment for pupils from poorer backgrounds.

In our analyses (Moore *et al.*, 2017), consistent with Markham and Aveyard (2003), we found that independent and interacting associations of school and family socioeconomic status observed for health behaviours were present for measures of subjective wellbeing, while these interactions were in part explained by a tendency for pupils from poorer backgrounds to perceive better relationships with school staff where they attended less affluent schools. Subsequently, Shackleton *et al.*(2018) used data from the INCLUSIVE trial in South‐East England to replicate our findings, incorporating a broader range of well‐validated measures. Notably, the finding in Wales that overall, pupils attending poorer schools tended to report higher levels of connectedness to school staff than those in more affluent schools was not replicated in the more highly marketised English school system, with the inverse association observed. Nevertheless, the authors replicated our findings that among pupils from poorer families, mental wellbeing and health‐related quality of life scores were lowest where they attended a school with a more affluent overall intake.

These studies point to the socioeconomic status of a school’s intake as a contextual characteristic which shapes the dynamics of school systems and influences pupil wellbeing in ways not fully explained by the affluence of individual pupils and families. This is consistent with the work of scholars such as Wilkinson and Pickett (2007), whose international comparative work has demonstrated the importance of thinking about socioeconomic status not strictly in terms of absolute deprivation, but as a relative and relational concept in which socioeconomic position relative to those by whom one is surrounded matters significantly for health and wellbeing.

## School transitions and mental wellbeing

Studies on the emergence of socioeconomic inequalities in health and wellbeing, including our own earlier analyses, have to date focused mostly on secondary schools. However, transition from primary to secondary school is a significant milestone in child and adolescent development (Coffey, 2013; van Rens *et al.*, 2018; Virtanen *et al.*, 2019), and a potential intervention point for reducing inequalities in health and wellbeing. In countries including the UK, most young people transition from a smaller primary school to a larger secondary school at 11 years of age. Most young people look forward to and adjust well to their new secondary school (Chedzoy & Burden, 2005); for many, moving to a larger school provides opportunities for interaction with a wider diversity of peers, and hence a greater likelihood of connecting with other individuals with similar interests. However, it can also bring challenges in relation to the loss of close relationships with primary school staff (Longobardi *et al.*, 2016), with young people often moving from a lot of time with a single teacher towards a smaller amount of time with a wider range of teachers. Transition to secondary school is commonly associated with dissolution of friendships and peer networks, with a minority of pupils maintaining the same ‘best friend’ through the transition to secondary school (Ng‐Knight *et al.*, 2019). Peer conflict and bullying may peak at the start of secondary school, as individuals attempt to assert their dominance among a new set of peers (Pellegrini & Long, 2002), prior to diminishing somewhat thereafter.

Life‐course mental health and wellbeing trajectories often have roots earlier in the life‐course (Riglin *et al.*, 2017), but the transition to secondary school coincides with a developmental period during which symptoms of mental health difficulties, such as depression and anxiety, commonly emerge (Viner *et al.*, 2015). School transition is therefore increasingly seen as a critical intervention point for minimising inequalities in educational attainment, for intervening to address the emergence of mental ill health at the individual level, and promotion of positive wellbeing at the whole‐school level (van Rens *et al.*, 2018; Virtanen *et al.*, 2019). Recent evidence shows that pupils who enter secondary school with depressive symptoms or conduct problems are less likely to perform well in terms of educational attainment (Riglin *et al.*, 2013), while vulnerable populations—such as children with autistic spectrum disorders—report particularly distressing experiences of school transition (Makin *et al.*, 2017). Studies on the impact of school transition on attainment have found the greatest drop in attainment through the transition amongst more socioeconomically deprived groups (Wilson, 2011; House of Commons, 2014), but fewer studies have focused on how socioeconomic status impacts mental wellbeing on transition to secondary school.

## Socioeconomic transitions in the movement from primary to secondary school

Young people’s management of the transition to secondary school is likely influenced by characteristics of the primary school which they leave, and the secondary school they join (Lester & Cross, 2015). To date, the relative contribution of the primary and secondary school context in explaining mental wellbeing through the transition has not received significant attention. A recent systematic review however emphasises a convergence of findings around the importance of positive parent–school–child relationships in ensuring a successful transition to secondary school (van Rens *et al.*, 2018). There is substantial evidence that these relationships vary by socioeconomic status; achieving high levels of parental engagement with schooling for example is commonly more challenging for schools in areas of deprivation (Goodall, 2017). For parents from poorer backgrounds whose children transition into a more affluent secondary school, relative social status within the more affluent networks of parents surrounding this new school may also impact on their own engagement with the norms of the school, exacerbating disconnections between school and family and contributing to inter‐generational reproduction of inequalities (Symonds, 2015). Hence, school transition may provide an important intervention point during which socioeconomic inequalities in wellbeing might be reduced, or indeed amplified. There is therefore substantial need to understand the independent and combined roles of primary and secondary schools in reducing (or amplifying) socioeconomic inequalities in wellbeing during this period.

In Wales and England, secondary schools are usually substantially larger than primary schools. For example, in 2018 the average state‐funded English primary school serves 250 pupils, while the average secondary school serves approximately 950 (National Statistics, 2018). Pupils from several primary schools therefore converge on a single secondary school. Given the narrower geographical regions served by primary schools, these will typically have greater between‐school dispersion in socioeconomic composition than secondary schools do. However, they will also have a greater degree of within‐school homogeneity. Hence, on transition to secondary school, a form of socioeconomic convergence occurs. Pupils from the most affluent ‘feeder’ primary schools transition into a school which is, on average, less affluent than their primary school. Conversely, pupils attending the least affluent feeder schools will typically transition into a school which is, on average, more affluent than their primary school. In both instances, pupils from primary schools at the more or less affluent end of the group of schools enter a secondary school which is more socioeconomically diverse than their primary school.

This socioeconomic convergence likely alters the dynamics of school settings in a number of important ways. First, pupils moving from poorer to more affluent schools are likely to be surrounded by a larger proportion of pupils from relatively affluent feeder schools. Hence, they are likely to assume a relatively disadvantaged position within their new school’s social hierarchy, amplifying challenges engaging with the instructional and regulatory order of the school (Markham & Aveyard, 2003), and the work young people engage in to hide their poverty from peers and school staff (Ridge, 2011). Second, for pupils from more homogeneously affluent primary schools, moving to a more socioeconomically diverse school likely increases their relative position within their school’s socioeconomic hierarchy. Through these dual processes, socioeconomic inequality in wellbeing may become amplified in the transition to secondary school.

This article draws on a combination of survey data from secondary schools throughout Wales, and routinely available data on the characteristics of young people’s primary and secondary schools to address the following key research questions:

- RQ1: What is the contribution of primary and secondary school contexts in explaining variance in young people's mental wellbeing in early secondary school years?
- RQ2: Is mental wellbeing following transition to secondary school associated with the *relative* socioeconomic composition of the young person's primary and secondary school?
- RQ3: Is the socioeconomic composition of the primary and secondary school attended by the young person independently associated with mental wellbeing?

## Method

### Sampling and data collection

Data were drawn from the 2017 School Health Research Network (SHRN) (Hewitt *et al.*, 2018; Murphy *et al.*, 2018) Student Health and Wellbeing (SHW) survey, completed by students in Years 7–11 from 193 secondary schools throughout Wales. Pupils at the start of their secondary school education (in Years 7 and 8; i.e. aged 11–13) were asked which primary school they attended prior to entry to secondary school, and therefore all analyses within this article focus on this subsample of pupils (*N* = 45,055). Schools joined the Network in three ways: those participating in the Welsh Health Behaviour in School‐aged Children (HBSC) survey in 2013/2014 were invited; nine schools in South Wales recruited to an HBSC sub‐study to pilot data linkage methods joined; and there were two rounds of open recruitment. The SHW survey is an online, closed response, self‐completion survey, available in English and Welsh. It measures self‐reported health behaviours among school students aged 11–16 years, and includes questions from the 2017/2018 Welsh HBSC survey with additional questions reflecting current policy, practice and research priorities in Wales. All network schools (*n* = 212, including every state‐maintained secondary school in Wales) were invited to participate in the 2017 SHW survey between September and December of the autumn term. This survey was administered using a web‐based interface by students during the school day. Participation was optional. Ethics approval was obtained from the Cardiff University School of Social Sciences ethics committee. Further information on survey methodology can be found via the national report available at www.shrn.org.uk/national‐data/.

### Measures

To measure mental wellbeing, we used the Short Warwick and Edinburgh Mental Wellbeing scale (SWEMWBS; Stewart‐Brown *et al.*, 2009), which has recently been successfully validated within the SHW survey (i.e. the dataset used for these analyses) for use with young people aged 11 or above (Melendez‐Torrez *et al.*, 2019). This includes seven positively worded statements (e.g. ‘I’ve been feeling optimistic about the future’, ‘I’ve been dealing with problems well’) and asks young people to indicate the box which best describes their experience of each statement in the past 2 weeks on a five‐point scale from ‘none of the time’ to ‘all of the time’. Pupils were asked in a free text field to enter the name of the primary school they attended, with spelling inaccuracies corrected by hand. In a small number of instances where children gave a school name which was linked to more than one primary school in Wales, we made an assumption that the primary school referred to was the one with greatest geographical proximity to the secondary school currently attended. Data on percentage of free school meal (FSM) entitlement of all primary and secondary schools were obtained from the StatsWales website (StatsWales, 2018). A transition score was calculated for each pupil by subtracting the FSM entitlement of their primary school from that of their secondary school, reversing such that an increased score represented an increase in school‐level affluence post‐transition, and dividing by 10 to facilitate interpretation of coefficients. All analyses with the exception of null models adjusted for sex (assessed by a single item asking young people to indicate whether they were a boy or a girl), ethnicity (assessed by a multi‐response question coded retrospectively as ‘white British’, ‘white other’ (including white Irish, white Gypsy Traveller and white other), or ‘minority ethnicity’ and family affluence. Individual socioeconomic status was assessed using the Family Affluence Scale (FAS) (Currie *et al.*, 2008), on which pupils respond to a series of questions on material affluence, including car and computer ownership, family holidays, bathrooms and dishwashers, as well as whether they have their own bedroom. These were summed to give an overall measure of family affluence. Scores range from 0 to 13 and were binarised at the median into two roughly evenly distributed groups, with scores of 10 and above representing pupils from more affluent families. This scale is derived from the international HBSC survey and typically achieves higher completeness and better validity than measures such as parental occupation with young people of school age (Currie *et al.*, 2008; Torsheim *et al.*, 2016).

### Statistical analyses

Our modelling strategy developed successively more complex models to understand how each factor of interest relates to mental wellbeing. All models were estimated as cross‐classified linear mixed‐effects models, with primary and secondary school as simultaneous levels. We first estimated a null variance components model to estimate individual, primary school and secondary school variance (Model 1), without adjustment for any individual or school‐level characteristics (RQ1). Successive adjustments in subsequent models enable us to further estimate the extent to which variance at the primary school level remains, or is explained, after adjustment for compositional and contextual characteristics of primary and secondary schools. We then accounted for compositional characteristics (sex, ethnicity, family affluence) as fixed effects in an adjusted variance components model (Model 2). We introduced the transition term as a fixed effect in Model 3. In Models 4a, 4b and 4c, we regressed school‐level intercepts for mental wellbeing on (a) primary school FSM entitlement, (b) secondary school FSM entitlement and (c) both primary and secondary school FSM entitlement, respectively. Hence, these models provide an estimate of the association of the transition in relative affluence between a child’s primary and secondary school, with mental wellbeing accounting for the deprivation level of the young person’s primary and secondary school independently and in combination. This enabled us to examine (i) whether the observed association of socioeconomic differences from primary to secondary school with mental wellbeing (RQ2) was driven by, or remained independent of, the deprivation level of the school from which the child transitioned and/or now attended and (ii) whether associations of primary and secondary school‐level deprivation with mental wellbeing were independent of one another (RQ3). All models used Markov chain Monte Carlo methods to facilitate estimation, as is standard for cross‐classified models, and were implemented in Mplus (Muthén & Muthén, Los Angeles, CA).

## Results

### Sample descriptions

Of the 45,055 young people in secondary school Years 7 and 8 who completed the SHW survey, 40,892 (90.8%) (mean (SD) of 212 (109) pupils per school) provided an answer to the question on which primary school they attended, including a total of 1,307 schools. Data on FSM entitlement were obtainable for 1,213 of the schools listed, with other schools attended by participating pupils having either closed and merged with other schools (hence not included in the school census which provided FSM data), or data on FSM being missing from the census. A total of 39,836 pupils attended schools for which FSM data were obtained. Primary schools attended by pupils had a mean FSM percentage of 16.1 (SD = 11.4; range 0.0–68.3%). Secondary schools (*N* = 185) had a similar average FSM (16.8%), though a less dispersed range (SD = 9.2; 2.5–50.5%). The mean transition score (i.e. the average increase in relative affluence level between primary and secondary school) was −0.6% (SD = 9.2), with a range from −39.4% to +55.9%. Mean FAS score was 9.3 (*N* = 38,576, SD = 2.3, range = 0–13). Complete data were provided on the SWEMWBS for 36,401 pupils, with a mean score for mental wellbeing of 22.6 (4.5). Overall, 19,929 (48.7%) identified as boys, 20,588 (50.4%) as girls, and 375 (0.9%) said that they did not want to answer. Most young people (*N* = 33,941; 83.0%) identified as ‘white British’, with 4.3% (*N* = 1,757) and 8.6% (*N* = 3,531) respectively categorised as ‘white other’ or ‘minority ethnicity’ and a further 1,663 (4.1%) declining to answer.

### What is the contribution of primary and secondary school contexts in explaining variance in young people's mental wellbeing in early secondary school years (RQ1)?

As indicated in the sequence of multi‐level models presented in Tables 1 and 2, while the vast majority of variance in mental wellbeing was at the individual level (i.e. approximately 97%), there was nevertheless a significant variance component at both the primary (0.46; 95% CI = 0.32–0.61) and secondary school levels (0.30; 95% CI = 0.20–0.40). In null models (i.e. those not containing any independent variables), a larger proportion of variance in mental wellbeing was observed at the primary school level than at the secondary school level (Model 1). Following adjustment for demographic composition of schools attended, variance at both the primary and secondary school level was somewhat reduced (Model 2). Significantly higher mental wellbeing was reported by boys, and pupils from more affluent families (Model 2). Mental wellbeing among ‘white other’ ethnicities was significantly lower than among ‘white British’ pupils, although no significant difference was observed between ‘white British’ and ‘minority ethnicity’ young people. Approximately a third of variance at the primary school level, and a sixth of variance at the secondary school level, was explained by compositional differences in socio‐demographics between schools, though this remained significant. Hence, this is consistent with a hypothesis that some of the variability between schools in mental wellbeing is explained by differences in their socio‐demographic composition, but that the primary school context from which a child transitions, and the secondary school context into which they transition, both influence pupil’s mental wellbeing.

**Table 1:** Individual and school‐level factors associated with young people’s wellbeing during the transition to secondary school

	Model 1: null model (*n* = 36,401)			Model 2: adjusted for individual demographics (*n* = 33,790)			Model 3: school transition (*n* = 32,283)		
Residual variance	26.28	0.19	[25.92, 26.66]	25.19	0.21	[24.75, 25.60]	25.18	0.19	[24.82, 25.57]
	Coeff.	SD	95% CI	Coeff.	SD	95% CI	Coeff.	SD	95% CI
Student‐level variables									
Sex									
Male				0.67	0.06	[0.55, 0.77]	0.66	0.05	[0.54, 0.75]
Ethnicity									
White non‐British				−0.86	0.13	[−1.10, −0.59]	−0.86	0.14	[−1.14, −0.60]
Minority ethnicity				−0.12	0.11	[−0.33, 0.10]	−0.09	0.11	[−0.30, 0.13]
Family affluence				1.10	0.06	[1.00, 1.21]	1.04	0.05	[0.94, 1.14]
FSM transition score							−0.23	0.04	[−0.30, −0.16]
School‐level residual variances									
Primary school residual variance	0.46	0.07	[0.32, 0.61]	0.32	0.04	[0.26, 0.41]	0.23	0.04	[0.15, 0.31]
Secondary school residual variance	0.30	0.05	[0.20, 0.40]	0.25	0.05	[0.18, 0.37]	0.27	0.05	[0.18, 0.39]

**Table 2:** Transition models for primary and secondary school entered separately and simultaneously (*n* = 32,283)

	Model 4a: transition, primary school random intercept			Model 4b: transition, secondary school random intercept			Model 4c: transition, primary, secondary random intercept		
Intercept	24.61	0.08	[24.48, 24.78]	24.57	0.08	[24.47, 24.76]	24.64	0.11	[24.42, 24.85]
Residual variances	25.19	0.19	[24.80, 25.56]	25.19	0.19	[24.80, 25.56]	25.19	0.20	[24.81, 25.58]
	Coeff.	SD	95% CI	Coeff.	SD	95% CI	Coeff.	SD	95% CI
Student‐level variables									
Sex									
Male	0.67	0.06	[0.56, 0.78]	0.67	0.05	[0.56, 0.77]	0.66	0.06	[0.55, 0.77]
Ethnicity									
White non‐British	−0.85	0.14	[−1.11, −0.59]	−0.85	0.14	[−1.11, −0.60]	−0.86	0.14	[−1.13, −0.58]
Minority ethnicity	−0.05	0.11	[−0.24, 0.15]	−0.06	0.10	[−0.25, 0.14]	−0.05	0.11	[−0.27, 0.16]
Family affluence	1.00	0.06	[0.89, 1.12]	1.00	0.06	[0.88, 1.12]	0.99	0.06	[0.88, 1.10]
FSM transition score	0.07	0.06	[−0.03, 0.19]	−0.28	0.04	[−0.35, −0.21]	−0.54	0.14	[−0.78, −0.27]
School‐level variables									
Primary school FSM	−0.36	0.05	[−0.45, −0.27]				0.25	0.14	[−0.01, 0.49]
Secondary school FSM				−0.33	0.04	[−0.41, −0.27]	−0.61	0.14	[−0.86, −0.37]
Residual variances									
Primary school residual variance	0.26	0.04	[0.18, 0.34]	0.26	0.04	[0.18, 0.34]	0.23	0.05	[0.14, 0.34]
Secondary residual school variance	0.18	0.04	[0.10, 0.27]	0.18	0.04	[0.10, 0.27]	0.19	0.04	[0.12, 0.29]

### Is mental wellbeing following transition to secondary school associated with the relative socioeconomic composition of the young person's primary and secondary school (RQ2)?

Where a term for transition is entered into models (Model 3), a significant negative association with mental wellbeing is observed (−0.23; 95% CI = −0.30 to −0.16). That is, where the child’s secondary school has a *more* affluent intake than their primary school, this is associated with *worse* mental wellbeing. Conversely, moving to a secondary school with a *lower* overall affluence (i.e. higher FSM entitlement) is associated with better mental wellbeing. Models 4a to 4c re‐examine this association with successive adjustment for the FSM entitlement of the child’s primary school, secondary school and both. In the final model (Model 4c), the association of school transition first observed in Model 3 is strengthened, with a doubling in coefficient (−0.54; 95% CI = −0.78 to −0.27). This indicates that a 10% decrease in FSM entitlement (i.e. an increase in affluence) in the young person’s secondary school (relative to the primary school from which they transitioned) is associated with an approximately half‐point reduction in mental wellbeing.

### Is the socioeconomic composition of the primary and secondary school attended by the young person associated with wellbeing (RQ3)?

In the models presented in Table 2, in which FSM entitlement for primary and secondary school are entered independently (Models 4a and 4b) and then together (Model 4c), both primary school (−0.36; 95% CI = −0.45 to −0.27) and secondary school (−0.33; 95% CI = −0.41 to −0.27) FSM entitlement demonstrates a similar degree of association with mental wellbeing. A more deprived overall composition is associated with worse mental wellbeing in both instances. More precisely, an increase of 10 percentage points in young people entitled to free school meals is associated with an average decline in mental wellbeing of approximately one‐third of a point. However, where combined into a single model (final model; Model 4c), the association of primary school FSM entitlement becomes non‐significant. This is consistent with a conclusion that associations of primary school‐level deprivation with mental wellbeing are explained via a tendency for pupils attending poorer or more affluent primary schools also to transition into relatively poor or affluent secondary schools. Associations of individually measured socioeconomic status (i.e. family affluence score) with mental wellbeing were not materially altered from Model 2 to subsequent models which included school‐level variables, consistent with a conclusion that associations of family and school‐level socioeconomic status with mental wellbeing are independent of one another; that is, the socioeconomic context of the school has an association with mental wellbeing which is not fully explained by the affluence of individual pupils’ families.

In the final model, significant variance remains at both primary (0.23; 95% CI = 0.14–0.34) and secondary school (0.19; 95% CI = 0.12–0.29) levels. However, this is reduced by approximately 40% relative to Model 1 (the null model) and by 25% relative to Model 2, which comprises only individual‐level variables. This is consistent with the conclusion that a substantial proportion of the variance between schools in mental wellbeing is explained by the contextual (school‐level) socioeconomic variables within the model.

## Discussion

This article has demonstrated via cross‐classified multi‐level analyses that in the first two years of secondary school, pupil mental wellbeing varies significantly according to both the primary school previously attended and the secondary school attended at the point of reporting. Indeed, residual variance at the primary school level was somewhat higher than that at the secondary school level in the null model, and models adjusting for only demographic differences. Hence, this is consistent with a conclusion of lasting primary school ‘effects’ on mental wellbeing, which endure beyond transition into secondary school, with the secondary school context also then contributing uniquely to variance in mental wellbeing. While a number of recent approaches have been effective in reducing the population level decline in mental wellbeing in the years after transition to secondary school (Bonell *et al.*, 2018), findings are consistent with a view that earlier intervention in primary schools is also needed to support positive wellbeing throughout adolescence (Riglin *et al.*, 2017). Indeed, while focused on prevention of mental illness rather than positive wellbeing, a small number of studies have demonstrated important benefits of universal primary school intervention prior to the transition to secondary school (Stallard *et al.*, 2014).

Where analysed in separate models, the socioeconomic composition of a pupil’s primary school, and that of their secondary school, exhibit similar correlation with pupil mental wellbeing in early secondary school years. That is, attending a poorer primary or secondary school predicted lowered mental wellbeing. Consistent with our analyses of earlier datasets in Welsh secondary schools, family‐level affluence remained a significant predictor of mental wellbeing throughout all models, consistent with a hypothesis of independent school and family socioeconomic effects on mental wellbeing (Moore & Littlecott, 2015; Moore *et al.*, 2017). Hence, this is consistent with the conclusion that a school’s socioeconomic composition impacts on the dynamics of the school context in ways which impact on pupil’s wellbeing, rather than associations of school‐level socioeconomic composition with mental wellbeing being fully explained via the affluence of individual pupils and their families. While cross‐classified to some extent, the compositional affluence of a secondary school in large part reflects an aggregation of the affluence levels of the schools feeding into it. Hence, where combined into a single model, only the term for secondary school FSM entitlement remained significant, likely due to substantial homogeneity in primary and secondary school socioeconomic contexts.

Our main research question focused on how socioeconomic transitions (i.e. movements to a more or less affluent school) impacted on wellbeing in the early years of secondary school. Previous literature has emphasised the importance of young people’s engagement with the instructional and regulatory order of their school, with greater commitment to the school community associated with better wellbeing outcomes (Markham & Aveyard, 2003). Consistent with psycho‐social theories of health inequality which emphasise the need to think of socioeconomic status in relative rather than absolute terms (Wilkinson & Pickett, 2007), our own previous work highlights the tendency for the lowest wellbeing where pupils from poorer backgrounds attend a relatively affluent school (Moore *et al.*, 2017). Hence, we theorised that movement from a more affluent to a less affluent school, or vice versa, would be associated with pupil mental wellbeing via impacts on pupils’ relative status within their new school’s socioeconomic hierarchy. We anticipated that pupils from poorer backgrounds transitioning into schools with higher socioeconomic intakes may experience a detriment to their wellbeing as a consequence of their lowered social status within their school, and vice versa. Consistent with our hypothesis, the affluence of a pupil’s secondary school, relative to their primary school, was significantly inversely associated with mental wellbeing. This association was maintained in all models, adjusting for primary school FSM entitlement, secondary school FSM entitlement and both, strengthening in magnitude in the final model. Our previous analyses have shown that the FSM entitlement of a secondary school predicts the largest portion of variance in pupil attainment between schools (Littlecott *et al.*, 2018). In this study, the discrepancy between the socioeconomic composition of a young adolescent’s primary and secondary school explained almost 30% of residual variance in mental wellbeing, after within‐school differences in socio‐demographics had been accounted for.

### Strengths and limitations

The study benefits from the use of a large nationally representative sample of schools and young people in Wales, and from the robust analytical approaches adopted. It uses a now well‐validated measure of young people’s mental wellbeing, and links this to objective sources of information on school characteristics. However, there are a number of important limitations and potential directions for future research. While multi‐level models adjusted for key demographic differences between schools, it is possible that school‐level variance may have been reduced further through inclusion of a broader range of indicators, such as between‐school differences in rates of parental mental ill health. As a consequence, school ‘effects’ may be somewhat overestimated. Further, mental wellbeing was not measured in primary school prior to the transition, and hence while we are able to draw conclusions regarding wellbeing in the early secondary school years and how this relates to school contexts, we are not able to longitudinally model change in individual pupils’ wellbeing trajectories through this transition.

### Conclusion

Clearly, schools are only one of many important influences on young people’s wellbeing and cannot fully compensate for structural inequalities and the unequal distribution of health outcomes. However, they have a role to play, and efforts to improve mental wellbeing through action within the education system need to understand the individual and combined influences of primary and secondary school contexts on pupils’ wellbeing. Attending a primary or secondary school with a poorer or more affluent overall intake may have effects on wellbeing which are independent of family affluence. With several small primary schools each converging on a larger secondary school, increases in relative affluence in the transition to secondary school will inevitably be disproportionately experienced by pupils moving from relatively poor primary schools and our analyses are, to our knowledge, the first to show that movement to a more affluent school is adversely associated with mental wellbeing. Hence, these findings position the transition to secondary school as a key point in the child’s school career, during which socioeconomic inequality in mental wellbeing is likely to widen. A combination of effective universal and targeted intervention in primary and secondary school, and at the point of transition, is likely needed to reduce these inequalities.

Important future directions for research include enhancing the understanding of the mechanisms through which socioeconomic transitions may impact upon adolescent mental wellbeing, in order to inform effective interventions to prevent the widening of socioeconomic inequality. We posit, for example, that consistent with Markham and Aveyard’s (2003) theory of human functioning and school organisation, the dynamics of school systems can positively or negatively influence wellbeing through their impact on pupils’ engagement with the instructional and regulatory order of the school. Previous research has highlighted the importance of comprehensive transition interventions that are multi‐faceted and long term, including involvement of parents and the creation of a sense of community and belonging to address the disjuncture between familial and impersonal environments (Anderson *et al.*, 2000). It is, however, plausible that parents whose children move into a more affluent school may also be less well connected to the school, due to their position within the socioeconomic hierarchies surrounding the school. Collection of data on mental wellbeing and hypothesised mechanisms underpinning a healthy transition (such as parental and pupil connectedness to school), and longitudinal analyses of how these factors predict change in mental wellbeing in transition to secondary school, would facilitate important extensions to these analyses, to inform effective intervention.

There is a current decline in coordinated transition interventions in some parts of the UK such as England, attributed to the increased pressure on schools to save money and the introductions of academies outside of local authority control (McLellan & Galton, 2015), and the likely impact of this disinvestment on the widening of inequalities deserves urgent attention. In Wales, where this research was conducted, it is mandated that all maintained secondary schools and their maintained ‘feeder’ primary schools must develop and publish a ‘transition plan’, outlining detailed and coherent arrangements to support transition (National Assembly for Wales, 2006; Estyn, 2010). To date, effective interventions which have been informed by the theoretical perspective on which we draw (Markham & Aveyard, 2003) have involved working with schools to identify ways in which current practice might be altered to optimise the health‐enhancing role of schools, with intervention about doing existing things better rather than more unsustainable approaches of adding ever more intervention into the work of crowded contexts such as schools (Bonell *et al.*, 2018). While schools have an important role to play in supporting young people’s wellbeing, poverty remains a structural issue requiring structural solutions, with schools one of many interconnected systems which impact young people’s wellbeing. Indeed, where schools do have a role in supporting wellbeing, making positive changes requires external support, including from higher policy levels within the education system. While in England, plans for education look likely to move towards harsher disciplinary structures which alienate many young people from school (Aditya *et al.*, 2019), ongoing curriculum reforms in Wales (Donaldson, 2015), within the context of the equity‐focused Wellbeing of Future Generations Act (National Assembly for Wales, 2015), includes a major focus on supporting the development of ‘healthy confident individuals’ as a core goal of the education system. These analyses point to school transition as an important area for research and action to achieve these goals.

## Funding

The School Health Research Network is a partnership between the Centre for the Development and Evaluation of Complex Interventions for Public Health Improvement (DECIPHer) at Cardiff University, the Welsh Government, Public Health Wales and Cancer Research UK, funded by Health and Care Research Wales via the National Centre for Health and Wellbeing Research. The work was undertaken with the support of DECIPHer, a UKCRC Public Health Research Centre of Excellence. Joint funding (MR/KO232331/1) from the British Heart Foundation, Cancer Research UK, Economic and Social Research Council, Medical Research Council, the Welsh Government and the Wellcome Trust, under the auspices of the UK Clinical Research Collaboration, is gratefully acknowledged.

## Conflict of interest

The authors declare that they have no conflict of interest.

## Data sharing statement

The data that support the findings of this study are available from the School Health Research Network upon reasonable request to shrn@cardiff.ac.uk. Some variables are restricted to preserve confidentiality.

## Ethical guidelines

Ethical approval was provided by the Cardiff University School of Social Sciences Research Ethics Committee. This study adheres to the ESRC framework for research ethics.
